# Influence of accessibility and distance in the consumption of disposable equipment in a hemodialysis unit

**DOI:** 10.1186/1472-6963-14-S2-P60

**Published:** 2014-07-07

**Authors:** Antonio Torres-Quintana, M Teresa Icart-Isern, Cristina Esquinas-López

**Affiliations:** 1 Unitat Hemodialisis. Fundació Puigvert, Barcelona, Spain; 2 School of Nursing, University of Barcelona, Barcelona 08907, Spain

## Background

The location of the disposable material in a hemodialysis unit is essential to ensure the effectiveness of the circuits and to provide quality in nursing care.

The law of the minimum effort may explain how the accessibility and distance of dressing trolleys can influence the consumption of some health care supplies [1,2]. It is necessary for health managers to look for effective strategies that optimize the use of wound care material without reducing the quality of care [1,3].

The objective was to determine whether the distance that nurses have to walk to access the trolley with the wound care material (gauzes, dressings and 10cc physiological serum), influence on the amount consumed in a hemodialysis unit at the Fundació Puigvert.

The research hypothesis is: consumption of those materials will decrease 5% as the distance to access the trolleys increases from 5 to 7 and from 7 to 9 meters.

## Materials and methods

Thirty nurses (one trolley each one) under two shifts participated in a quasi-experimental design. For two months, trolleys were placed 5 meters away from the patient bed, another two months they were placed 7 meters and in the last two months the distance was 9 meters.

## Results

Significant differences were observed comparing the consumption of physiological saline solution, gauzes and dressings when trolleys were located at 7 meters versus the 5 meters (being lower consumption, p<0.001) and when they were located from 7 to 9 meters (consumption being lower, p<0.001). There was an inverse linear relationship between the consumption of gauze and dressings and experience of nurses for any distance (RR = 1.26-0.112 duration; p=0.03).

## Conclusions

Correlation between consumption of material and age and experience was found inverse for all distances. A lower consumption of material by older and more experienced nurses was found. The location of material leads to a reduction in costs without any consequence in quality of care for patients in a hemodialysis unit.
